# Pharmacodynamics and Cellular Uptake of Peimine and Peiminine in Inflammatory Model Non-Small-Cell Lung Cancer Epithelial Cells (A549)

**DOI:** 10.1155/2022/2946201

**Published:** 2022-02-07

**Authors:** Zhan-Ke Chen, Di Zhao, Su-Xiang Feng, Jiangyan Xu

**Affiliations:** ^1^Shandong University of Traditional Chinese Medicine, Jinan, China; ^2^The First Affiliated Hospital of Henan University of Chinese Medicine, Zhengzhou 450000, China; ^3^Henan University of Chinese Medicine, Zhengzhou 450046, China; ^4^Henan Key Laboratory of Chinese Medicine for Respiratory Disease, Henan University of Chinese Medicine, Zhengzhou, Henan 450046, China; ^5^Co-Construction Collaborative Innovation Center for Chinese Medicine and Respiratory Diseases By Henan & Education Ministry of P.R. Zhengzhou, Zhengzhou 450046, China

## Abstract

Peimine and peiminine are isosteroidal alkaloids with multiple biological activities, such as anticancer and anti-inflammatory activities, but their cellular uptake and pharmacodynamics are unclear. In this study, a rapid and sensitive ultra-performance liquid chromatography-tandem mass spectrometry (UPLC-MS/MS) method was developed for the simultaneous quantification of peimine and peiminine concentrations in A549 cells. In the pharmacodynamic study, the selected inflammatory cytokines were IL-8, MMP-9, and TIMP-1. The results demonstrated that all calibration curves exhibited good linearity (*r* > 0.9970). The RSDs of intraday and interday precision and accuracy were less than 6.73% and 1.76% and 7.73% and 3.05% for peimine and peiminine, respectively. Moreover, the average analytic recoveries ranged from 83.85% to 113.67%, and the matrix effect was within 95.05%–111.29%. The uptake experiment showed a time-dependent characteristic in the A549 cells. The combination group had increased uptake and had a longer *T*_max_ than the single group. In the experimental pharmacodynamics groups, the anti-inflammatory effects of the 100.0 µg/mL combination group were the most obvious. This investigation, for the first time, explores the cellular uptake profiles and pharmacodynamics of peimine and peiminine in A549 cell lines.

## 1. Introduction

Targeted drug therapies are receiving more and more attention. Cellular uptake plays an indispensable role in the research of drug disposal in cells and in predicting and evaluating drug efficacies. Peimine and peiminine are isosteroidal alkaloids and are the main biologically active components of Fritillariae Thunbergii Bulbus (FTB) [1]. FTB is a significant Chinese medicine that has been clinical for thousands of years, with bitter and cold properties that clear heat, resolve phlegm, relieve cough, detoxify, and eliminate carbuncles [2, 3]. Modern pharmacological studies show that peimine and peiminine display analgesic [4], anti-inflammatory [5], and antitumor [6] biological activities. They also relax smooth muscles [7]. Peimine significantly inhibits the secretion of proinflammatory cytokines (e.g., TNF-*α*, IL-6, and IL-1*β*), increases anti-inflammatory cytokines (e.g., IL-10), and inhibits the production of lipopolysaccharide (LPS) induced inflammatory cytokines. It blocks the signaling pathways of the extracellular signal-regulating kinase (MAPK) and nuclear factor-kappa B (NF-kB) [8, 9]. Peiminine reportedly has a strong anti-inflammatory effect on a variety of diseases, making it a potential therapeutic drug for pulmonary diseases [10], osteoarthritis (OA) [11], Parkinson's disease (PD) [12], and mast cell-associated allergic inflammatory diseases [13]. In mouse OA chondrocytes, peiminine reduces IL-1*β* by inhibiting AKT/NF-kB and activating Nrf2/HO-1 to induce the inflammatory response [11]. Also, peiminine inhibited neuroinflammation to protect dopaminergic neurons in the LPS-induced PD rat model and significantly reduced the production of proinflammatory mediators in BV-2 cells [12].

Cellular pharmacokinetics is an emerging branch of classical pharmacokinetics that has received extensive attention for its role in drug evaluation and development in recent years [14]. A growing number of reports indicate that pharmacokinetic research based on plasma drug concentrations cannot fully elucidate the pharmacological effects of drugs in some tissues (e.g., tumors or brain tissues). Furthermore, it is difficult to truly and effectively predict drug efficacy in vivo [15]. The drug concentration around the intracellular target can provide a truer reflection of drug efficacy since the correlation between the drug concentration in the cell and the toxic reaction is stronger than in the plasma [16–18]. This is a microscopic perspective of pharmacokinetics that considers the cell as a whole to quantitatively analyze the dynamics of drug uptake, distribution, metabolism, and excretion within the cell to assess the efficacy of the drug in the target cell [19]. At present, there have been studies on the pharmacokinetics of peimine and peiminine in rats and beagles [20, 21]. Cellular uptake is one of the central challenges in chemical biology and beyond. With the objective to find conceptually innovative ways to enter cells, cyclic oligochalcogenides (COCs) are emerging as powerful tools [22]. Cellular uptake uses live cells to examine the effects of compounds on receptor or transporter systems. It can determine a compound's potency and efficacy. It can also be used to measure a compounds transport into cells or efflux from cells. Cell experiments in vitro have shown that peimine displays anti-inflammatory and analgesic properties at the cellular level [5]. No method has been reported for the simultaneous determination of peimine and peiminine in cell samples, nor for its application in cellular uptake studies, and only a few studies have been combined with pharmacodynamics.

A549 cells, characterized by alveolar type II epithelial cells, are the preferred cells for establishing in vitro cell models of acute lung injury [23]. TNF-*α* stimulates A549 cells to induce an inflammatory response, which may be related to the activation of excessive protease, the increase of interleukin levels, and the induction of airway epithelial cells into goblet cells, increasing inflammatory gene expression [24]. Thus, we used pharmacodynamics to study the effects of peimine and peiminine o TNF-*α* induced A549 cell inflammation models using the inflammatory cytokines IL-8, MMP-9, and TIMP-1. In addition, we developed and validated a specific and sensitive UPLC-MS/MS method for the simultaneous determination of peimine and peiminine concentrations in A549 cell lysate and successfully applied this method to the study of cellular pharmacokinetic characteristics. This paper provides a useful reference of the pharmacologic mechanisms and dynamic laws in cells and a theoretical basis for the safe and effective use of peimine and peiminine.

## 2. Materials and Methods

### 2.1. Material and Reagents

Peimine and peiminine were purchased from the National Institutes for Food and Drug Control (Beijing, China). Carbamazepine (internal standard; IS) was obtained from Shanghai Yuan Ye Bio-Technology Co., Ltd. (Shanghai, China). These reference substances had a purity of >98%. The raw material peimine (purity ≥ 90%) was obtained from Xi'an Hui Lin Bio-Technology Co., Ltd. (Shaanxi, China). The raw material peiminine (purity ≥ 90%) was provided by Chengdu Master Biotechnology Co., Ltd. (Chengdu, China). Methanol and acetonitrile were provided by the Tedia Company (USA). Ammonium formate was obtained from Fisher (USA). All solutions were mass spectrum grade. Fetal bovine serum, trypsin, methyl thiazole tetrazolium (MTT), and Roswell Park Memorial Institute (RPMI) medium 1640 were purchased from Beijing Solarbio Science and Technology Co., Ltd. (Beijing, China). TNF-*α* was purchased from Peprotech (USA). The ultrapure water was produced by the Millipore Mill-Q system (Bedford, MA, USA). Human IL-8, MMP-9, and TIMP-1 ELISA kits were purchased from Baxter Biological Co., Ltd. The A549 cell line was obtained from Shanghai Institutes for Biological Sciences (Shanghai, China).

### 2.2. Cell Culture

A549 cell lines were cultured in RPMI 1640 medium that was supplemented with 10% fetal bovine serum (FBS) and then incubated at 37°C under a humidified incubator of 5% CO_2_. The cell culture medium was changed every 2 days and passaged every 3 days at a ratio of 1 : 3. The A549 cells grew in a logarithmic period and were mixed into 2 × 10^6^/ml in the cell culture medium. After the cells were mixed with cryopreservation solution (DMSO : FBS: RPMI Medium 1640 (1 : 2 : 7)), they were kept at 4°C for 2 h, followed by −20°C for 6 h, and then stored in liquid nitrogen.

### 2.3. MTT Assay for Cell Viability

A549 cells were seeded at a density of 2 × 10^4^/mL in a 96-well ordinary flat-bottom plate at 100 *μ*L per well. Once the cells reached 80%, they were grouped as follows: blank group (K, 10% FBS complete medium), model group (M, containing 10 ng/mL TNF-*α*, 10% FBS complete medium), 12 normal cell experimental groups (4 different peimine concentrations, 4 different peiminine concentrations, and 4 different peimine + peiminine concentrations), and 12 model cell experimental groups (4 different peimine concentrations, 4 different peiminine concentrations, and 4 different peimine + peiminine concentrations). The concentrations of peimine and peiminine in each of the 4 groups were 25 *μ*g/mL, 50 *μ*g/mL, 100 *μ*g/mL, and 200 *μ*g/mL. The culture plates were placed in an incubator, and the supernatant was aspirated after 24 h and then added to 100 *μ*L of the medium containing MTT (0.5 mg/ml) for 4 h. Next, the supernatant was aspirated, and 150 *μ*L DMSO was added. The solution was shaken for 10 min in each well. Finally, the absorbance (OD value) was measured at a wavelength of 490 nm, and the cell survival rate was calculated.

### 2.4. Pharmacodynamics Study

#### 2.4.1. Grouping

The blank group (K) was grown in 10% FBS complete medium. The model group (*M*) contained 10 ng/mL TNF-*α* in the 10% FBS complete medium. The 12 model experimental groups include: 4 peimine concentrations (12.5 *μ*g/mL, 25.0 *μ*g/mL, 50.0 *μ*g/mL, and 100.0 *μ*g/mL); 4 peiminine concentrations (12.5 *μ*g/mL, 25.0 *μ*g/mL, 50.0 *μ*g/mL, and 100.0 *μ*g/mL); 4 peimine + peiminine concentrations (peimine : peiminine = 1 : 1, peimine: 12.5, 25.0, 50.0, and 100.0 *μ*g/mL; peiminine: 12.5, 25.0, 50.0, and 100.0 *μ*g/mL).

#### 2.4.2. Index Detection

The A549 cells were seeded at a density of 2 × 10^6^ cells per well in a 6-well culture plate at 2 mL and then administered 24 h later. The supernatant culture solution was collected by centrifugation 48 h after dosing and then stored at −80°C. The cells were then lysed by adding 300 *μ*L of RIPA cell lysate per well. The lysate was collected 10 min later. The supernatant was collected after centrifugation of the lysate, and proteins were detected using the BCA method. The contents of IL-8, MMP-9, and TIMP-1 in the supernatant were determined using an ELISA kit according to the manufacturer's instructions.

### 2.5. Cellular Uptake Study

#### 2.5.1. Apparatus and Operation Conditions

An Ultimate3000 HPLC system (Thermo Scientific, San Jose, USA) equipped with a quaternary pump, an online degasser, an autosampler, a column temperature compartment, and a UV detector was utilized for LC separation. An XBridgeTMC_18_ (2.1 mm × 150 mm, 5 *μ*m) was employed at 30°C. The mobile phase consisted of acetonitrile (A) and 10 mmol/L ammonium formate (B; 30 : 70) that was delivered at a flow rate of 0.3 mL/min.

The Q Exactive mass spectrometer (Thermo Scientific, San Jose, USA) equipped with a heat electrospray ionization source operated in the full scan mode was used for MS. The carrier gas was nitrogen, and the pressure of the sheath gas and the auxiliary gas were 35 bar and 10 bar, respectively. Positive and negative ions were scanned simultaneously, and the full scan range was from 150 to 1500 *m*/*z*, with the first-order resolution of 70,000. The spray voltage was +3.5 kV or −2.8 kV under the positive or negative mode, respectively. The auxiliary gas heater and capillary temperatures were maintained at 200°C and 350°C, respectively. The ions to be measured and labeled for quantitative analysis were peimine, m/*z* 432.3472 [*M* + *H*]+, peiminine, *m*/*z* 430.3316 [*M* + *H*]+, and carbamazepine, *m*/*z* 237.1022 [*M* + *H*]+.

#### 2.5.2. Cell Sample Preparation

Briefly, the collected cells were placed in a centrifuge tube and centrifuged for 5 min at 1 × 10^4^ r/min. The supernatant was discarded, and 300 *µ*L ultrapure water was added to each tube. After mixing, the solutions underwent 5 freeze-thaw cycles (−80°C/37°C), followed by ultrasonic treatment for 30 min, and then were centrifuged at 12000 r/min for 20 min. The supernatant was placed into new centrifuge tubes at 2.5 µL each to determine protein concentrations via the BCA method. The remaining supernatant was added to 100 *µ*l IS and 1200 *µ*l methanol [25]. Then, it was shaken for 15 min to mix thoroughly and centrifuged at 12000 r/min for 20 min. Next, the supernatant was concentrated via centrifuge, and the residue was dissolved in 100 *μ*L of the initial mobile phase to prepare a solution for the experiment. A total of 5 *μ*L of the prepared test solution was used for UPLC-MS detection. The final cell uptake was measured by intracellular protein content (drug (ng)/protein (*μ*g) [26], determined three times in parallel with each cell group. The freeze-thaw cycles were repeated at −80°C and 37°C and were stored at each temperature for 5 min. The ultrasonic lysis had a power of 250 W and a frequency of 100 KHz, with continuous ultrasound. Three parallel samples were taken at each time point, and each sample was injected three times with an injection volume of 5 *μ*L, which was calculated using the IS method.

#### 2.5.3. Validation of the UPLC-MS/MS Method

Methodological validation conformed to the guidelines for bioanalytical method validation issued by the FDA Center for Drug Evaluation and Research. [27].

*(1) Selectivity*. The logarithmic phase A549 cells were seeded at a density of 2 × 106 cells in a 10 cm diameter Petri dish and were cultured overnight in a CO_2_ incubator. Then, cells were divided into two group: the blank group and the administered group. The administered group cells were simultaneously added with peimine and peiminine and then incubated for 240 min at a final concentration of 100 *μ*g/mL. At the end of incubation, the cells were washed four times with PBS. Afterward, trypsin was added to each culture dish to digest the cells, and then the cells in each culture dish were collected and placed in a corresponding centrifuge tube. The intracellular drug concentrations were determined according to the sample preparation method under Section 2.5.2. The cells in each group were measured in parallel three times. The blank cell lysate, cell lysate containing peimine and peiminine, standard peimine and peiminine, and blank methanol were subjected to LC-MS detection according to Section 2.5.1.

*(2) Linearity and LOQ*. The cells were collected, and a series of cell samples with different concentration were prepared by adding a series of mixed control solutions of 100 µL peimine and peiminine and 100 *μ*L internal standard carbamazepine reference solution (249.6 ng/mL) using the sample treatment method under Section 2.5.2. According to the HPLC-MS operation conditions under Section 2.5.1, the concentration of the measured substance in the cell (ng/mL) was used as the horizontal coordinate. The actual peak area ratio of the measured substance in the cell and the IS was used as the vertical coordinates. The weight of 1/*X* was taken as the linear regression calculation to obtain the regression equation to establish the standard curve. The minimum detection limit (LOD) was measured with a signal-to-noise ratio (*S*/*N*) of 3 : 1, and the minimum quantitative limit (LOQ) was measured with the signal-to-noise ratio (*S*/*N*) of 10 : 1.

*(3) Extraction Recovery and Matrix Effect*. The extraction recovery and matrix effect were assessed with six replicates of QC samples at three levels. The extraction recovery was calculated by comparing the mean peak areas of the blank cell lysate with the analytes spiked before and after extraction. The matrix effect was evaluated as the ratio of the peak areas obtained from the cell lysate samples spiked with analysts after extraction with the standard solutions at the corresponding concentration.

*(4) Accuracy and Precision*. The intra- and interday accuracy and precision were assayed by analyzing six replicates of QC samples at three concentration levels (low, medium, and high) on the same day and on three consecutive days. The relative error and relative standard deviation (RSD) were calculated.

*(5) Stability*. Six replicate QC samples prepared in parallel at high, medium, and low concentration levels were exposed to various conditions to study sample stability. The short-term stability was carried out by storing the QC samples in the autosampler (4°C) for 24 h. The long-term stability was performed by storing the QC samples at −80°C for 30 days. For freeze-thaw stability, the QC samples underwent three complete freeze/thaw cycles at −20°C–25°C.

#### 2.5.4. Incubation Time and Uptake

The logarithmic phase A549 cells were seeded at a density of 2 × 10^6^ cells in a 10 cm diameter Petri dish at 37°C in a 5% CO_2_ incubator and then cultured for 24 h. Afterward, the supernatant was removed, and the cells were assigned to the following groups: normal peimine group, normal peiminine group, normal combination group, model peimine group, model peiminine group, and model combination group. The peimine and peimine concentrations were 100 *μ*g/mL. The action time of the drug was 10, 30, 45, 60, 90, 120, 180, 240, 360, and 480 min. The drug was removed at the end of the corresponding action times. After incubation, the cells were washed four times with PBS solution in the plate. The cells in each well were collected and placed in a centrifuge tube after trypsin digestion, and the samples were prepared using the method under Section 2.5.2.

#### 2.5.5. Release Assay

The logarithmic phase A549 cells were seeded at a density of 2 × 10^6^ cells in a 10 cm diameter Petri dish and cultured at 37°C in a 5% CO_2_ incubator for 24 h. Afterward, the supernatant was removed. For drug release measurements, a certain concentration of peimine and peiminine was spiked to make the final concentration of the drug 100 *μ*g/mL in the peimine and peiminine administration groups and then incubated for 120 min and 60 min, respectively. Meanwhile, a certain concentration of mixed solution was added to make the final concentrations of peimine and peimine in the combined administration groups 100 *μ*g/mL and then incubated for 240 min. At the end of incubation, the cells were washed four times with PBS and then incubated for 0, 10, 30, 45, 60, 90, 120, 180, 240, 360, 480, 720, and 1440 min in a fresh culture medium and placed in the incubator. Afterward, the cells were washed four times with PBS solution. Then, trypsin was added to each culture dish to digest the cells. Finally, the cells in each culture dish were collected and placed in a corresponding centrifuge tube. The cellular drug concentrations were determined according to the method under Section 2.5.2. Each group was measured in parallel three times.

### 2.6. Statistical Methods

Statistical analysis was calculated using SPSS19.0 software. Mapping analysis was performed using GraphPad Prism 7.00 drawing software. The values were expressed in terms of mean ± standard deviation. Differences were compared between the groups using one-way ANOVA. *P* value <0.05 indicated statistically significant differences. *P* values <0.01 were considered significant.

## 3. Results

### 3.1. Cell Viability Assays

**Figure 1:**
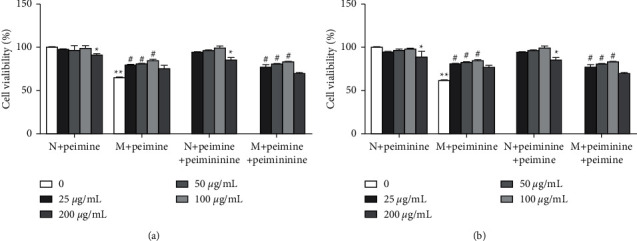
Effect of different concentrations of peimine and peiminine on the viability of A549 cells. (a):*N* + peimine: normal group (0 *μ*g/mL), normal single groups (25, 50, 100, and 200 *μ*g/mL); *M* + peimine: model group (0 *μ*g/mL), model single groups (25, 50, 100, and 200 *μ*g/mL); *N* + peimine + peiminine: normal combination groups (25, 50, 100, 200 *μ*g/mL; *M* + peimine + peiminine: model combination groups (25, 50, 100, and 200 *μ*g/mL). (b): *N* + peiminine: normal group (0 *μ*g/mL), normal single groups (25, 50, 100, 200 *μ*g/mL); *M* + peiminine: model group (0 *μ*g/mL), model single groups (25, 50, 100, and 200 *μ*g/mL); *N* + peiminine + peimine: normal combination groups (25, 50, 100, and 200 *μ*g/mL); *M* + peiminine + peimine: model combination groups (25, 50, 100, and 200 *μ*g/mL). The concentrations of peimine and peiminine in the combination groups were as follows: peimine:peiminine = 1 : 1, peimine: 25, 50, 100, and 200 *μ*g/mL and peiminine: 25, 50, 100, and 200 *μ*g/mL (^*∗*^*P* < 0.05 and ^*∗∗*^*P* < 0.01 mean significant difference compared with the normal group, and ^#^*P* < 0.05 means significant difference compared with the model group).

As shown in Figure 1, there were significant differences in cell viability between the model blank cell group and the normal blank cell group (*P* < 0.01), indicating that the A549 cell model was established successfully. Compared to the model blank group, the model single group and the model combination group showed a significant increase in cell viability with an increasing trend when the concentrations of the peimine and peiminine were 25–100 *μ*g/mL (*P* < 0.05).

### 3.2. Pharmacodynamic Results

**Figure 2:**
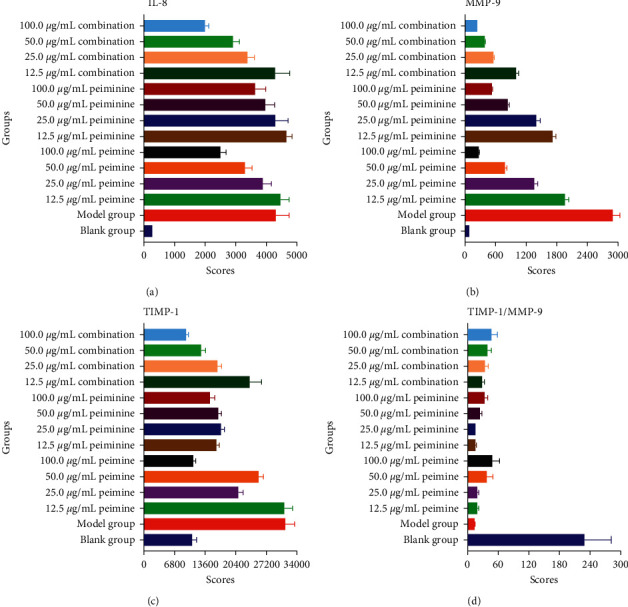
Inflammatory cytokines: (a) IL-8, (b) MMP-9, (c) TIMP-1, and (d) TIMP-1/MMP-9.

The interleukin-8 (IL-8), matrix metalloprotein-9 (MMP-9), and tissue inhibitor of metalloproteinases-1 (TIMP-1) in the blank group, the model group, and the 12 experimental groups are summarized in Table 1 and Figure 2. Compared to the blank group, the inflammatory cytokines (IL-8, MMP-9, and TIMP-1) were significantly increased, whereas TIMP-1/MMP-9 was significantly decreased (*P* < 0.01) in the model group. These results showed that the A549 cellular inflammatory model was established successfully and that the pharmacodynamic characteristics could be reflected with the three inflammatory cytokines. Meanwhile, IL-8 was significantly decreased in the 50.0 *μ*g/mL combination and the 100.0 µg/mL combination groups. In addition, the highest efficacy was seen in the 100.0 *μ*g/mL combination group, which showed a significant reduction in the three inflammatory cytokines and an increase in TIMP-1/MMP-9.

### 3.3. Cellular Uptake Studies

#### 3.3.1. Screen Optimal Dosing Concentration by MTT Colorimetry

**Figure 3:**
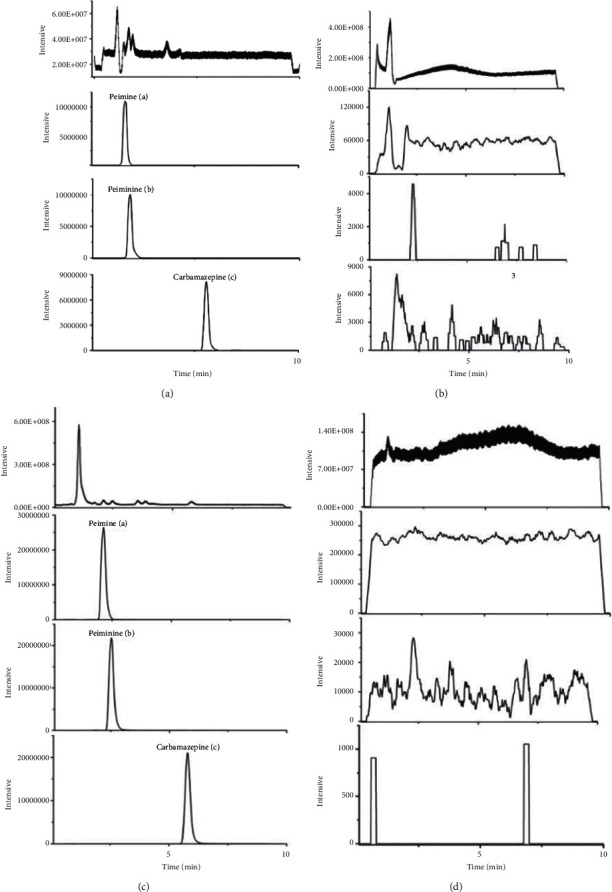
Representative chromatograms of peimine (a), peiminine (b), and IS (c): (a) chromatogram of mixed reference; (b) chromatogram of blank methanol; (c) chromatogram of cell extracts containing drugs; (d) chromatogram of blank cell.

The final concentration of peimine and peiminine in the cellular pharmacokinetic experiment was 100 *μ*g/mL according to the cell viability assay and pharmacodynamic results (Figure 3).

#### 3.3.2. Validation of the UPLC-MS/MS Method

*(1) Selectivity*. The retention time of the drug-containing cell lysates was consistent with that of standard substances. There were no interference peaks in the blank cell lysates to peimine and peiminine, and high responsiveness and good separation were achieved.

*(2) Linearity and LOQ*. The linear ranges of peimine and peiminine were 0.4008–2004 ng/mL and 0.2840–1420 ng/mL, respectively. The correlation coefficient (*r*) was greater than 0.9970. The minimum detection limits for peimine and peiminine were 0.1982 and 0.0951 ng/mL, respectively, which ensured the accurate determination of low concentrations in the cell samples (Table 2).

*(3) Extraction Recovery and Matrix Effect*. The matrix effects were 95.05%–111.29%, indicating that the cell matrix did not affect the quantitative detection of peimine and peiminine. The extraction recovery rates were 83.85%–113.67%, indicating that the experimental method was good (Table 3).

*(4) Accuracy and Precision*. As shown in Table 4, the intra- and interday precision of peimine and peiminine were 2.14%–6.73% and 4.33%–7.73%. The intra- and interday accuracy were −1.29%–1.76% and −0.22%–3.05%, thus satisfying the requirements.

*(5) Stability*.  Table 5 summarizes the stability evaluation results of the analysts under the different storage conditions. The measured concentration deviations of the quality control samples were less than ±15% of its nominal values after treatment under different conditions. This indicates that the method had satisfactory stability for the determination of peimine and peiminine and that it was suitable for sample analysis.

#### 3.3.3. Incubation Time and Uptake

**Figure 4:**
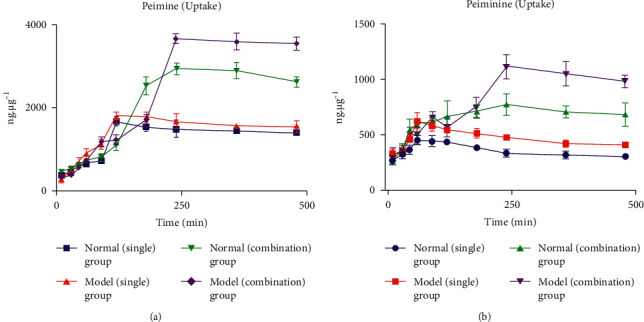
Incubation time and uptake curves (mean ± SD, *n* = 3): (a) peimine; (b) peiminine

The relationship between incubation time and the cellular uptake of peimine and peiminine was quantified by measuring the cellular concentrations at various time points after cell lysis, as presented in Table 6. As shown in Figure 4, the uptake characteristics of peimine and peiminine in the A549 cells were significantly different. In the single group, the peak times (*T*_max_) of the cell uptake of peimine and peiminine were 120 min and 60 min, respectively. In the combination group, *T*_max_ of peimine and peiminine were 240 min. In the combination group, the maximum intake (*I*_max_) of peimine and peiminine was significantly higher than in the single administration group. *I*_max_ of peimine and peiminine was higher in the model combination group than the normal combination group.

#### 3.3.4. Release Assay

As presented in Table 7, the relationship between the release time and intracellular uptake of peimine and peiminine was assessed using the intracellular drug concentrations after cell lysis. The calculated mean intracellular drug concentrations of peimine and peiminine were plotted as different times after withdrawal for each group. In the normal (single) group, model (single) group, normal (combination) group, and model (combination) group, both peimine and peiminine were rapidly released in the cells.

## 4. Discussion

The precipitation protein method (PPT) was selected for sample pretreatment in this study, because PPT is economical, practical, and simple to perform [28]. At present, the commonly used reagents for protein removal are methanol, acetonitrile, chloroform, and ethyl acetate. In this experiment, the matrix effect and extraction recovery rates of peimine, peiminine, and IS were investigated under methanol and acetonitrile (1 : 1) and cell dosage 4 : 1. The results showed that pure methanol could remove the proteins with less interference than the other two solvents and that the matrix effects were 75%–115%, with recovery rates of greater than 70%. In the evaluation of the 4 mobile phase systems, methanol water (A), acetonitrile water (B), acetonitrile 0.1% formic acid water (C), and acetonitrile-10 mmol/mL ammonium formate (D), it was shown that the method selected (D) to determine the peimine and peiminine and to separate the components to be tested and IS was good. The interference of the matrix was lower.

Inflammation is the body's natural defense response to various injury factors and the common pathological process in various diseases, which involves multiple mechanisms and pathways. Matrix metalloproteinase inhibitors (TIMPs) are endogenous natural inhibitors of MMPs, which can form complexes (1 : 1 binding) with MMPs to inhibit them [29]. Thus, three inflammatory cytokines and TIMP-1/MMP-9 were considered as a whole. The anti-inflammatory effects of the 100.0 *μ*g/mL combination group were the most obvious of the 12 experimental groups (删除, 改为: compared with the model group, the MMP-9 and TIMP-1 of peimine and peiminine of the 100.0 µg/mL showed significant difference (*P* < 0.01). There are significant differences of IL-8, MMP-9, and TIMP-1 between the model group and the combination group (*P* < 0.01). Interestingly, the anti-inflammatory effects are more attributed to peimine than peiminine). Peimine showed a dose-dependent inhibition of MMP-9 secretion in the concentration range of 12.5–100 µg/mL and significantly decreased IL-8 secretion at concentrations of 50 *μ*g/mL and 100 µg/mL. TIMP-1 secretion of peimine was significantly decreased at concentrations of 100 *μ*g/mL, while the ratio of TIMP-1/MMP-9 was significantly increased at 100 *μ*g/mL. In addition, peiminine also inhibited the secretion of MMP-9 in a dose-dependent manner and significantly inhibited the secretion of TIMP-1 in a concentration range of 12.5–100 *μ*g/ml. At the doses of 25 *μ*g/mL and 50 *μ*g/mL, the amount of TIMP-1 secreted did not change significantly, indicating that the inhibitory effect of peiminine was at a plateau stage, which differed from previous studies [30].

Considering the cellular uptake of peimine and peiminine, their final concentrations of 100 *μ*g/mL were screened using the MTT colorimetric method and combined with the pharmacodynamic results. Next, the relationship between incubation time and peimine and peiminine uptake was investigated. The results showed that the uptake of peimine increased with prolonged incubation time within 120 min in the single groups. Among them, the normal group showed that the uptake of peimine started to increase rapidly at 90 min and then decreased slowly after 120 min. In the range of 180–480 min, a steady state was reached. The model group showed that the uptake of peimine began to increase rapidly at 45 min, with a change range of 240–480 min that was not significant, and reached a steady state. In the combination group, the uptake of peimine increased with the prolonged incubation time within 240 min. In the single group, *T*_max_ of peiminine was 60 min, whereas the combination group had a peiminine *T*_max_ that was the same as that of peimine at 240 min. In the normal single group and the model single group, *I*_max_ of peimine was 1667.58 ± 109.83 and 1823.28 ± 97.18 ng/µg, respectively, with no statistical difference (*P* > 0.05). *I*_max_ of peiminine was 449.07 ± 39.65 and 622.72 ± 72.70 ng/*μ*g, with significant differences (*P* < 0.05*P* < 0.05). *I*_max_ of peimine and peiminine in the normal combination group were 2933.14 ± 126.76 and 765.37 ± 99.30 ng/*μ*g, respectively, which were significantly higher than that of the normal single group (*P* < 0.01). *T*_max_ of peimine and peiminine were prolonged in both the normal and model combination groups, which may be due to the similar structure of peimine and peiminine, indicating that the drug had some competitive effects in the process of entering cells. Compared with the single group, the combination group had a significantly increased uptake of peimine and peiminine, which may be due to the synergistic effect of peimine and peiminine to change some cellular mechanisms and increase cellular drug uptake, but this phenomenon needs further research (删除,改为: in the model cells, *T*_max_ of uptake of peimine and peiminine were 120 min and 60 min with the combination group 240 min. *T*_max_ were prolonged significantly in combination group (*P* < 0.01). The same trend was seen in normal cells. The reason was speculated that because of the the similar structure of peimine and peiminine, there may be a competitive effect in the process of uptake. In the experiment involving the intracellular release of peimine and peiminine, the normal single group, the model single group, the normal combination group, and the model combination group rapidly eliminated peimine and peiminine in the cells, with a maximum removal rate of 30 min, and the eliminating trend was the same. This may be related to how the drug was discharged intracellularly and the similar structure of peimine and peiminine, indicating that A549 cells had strong release abilities to peimine and peiminine in both normal and model conditions.

Combined with previous pharmacodynamic and uptake studies of peimine and peiminine at the cellular level, this study showed that the pharmacodynamic strength typically relates to its uptake in the cell. In this experiment, the concentrations of peimine and peiminine were 100 *μ*g/mL, *I*_max_ of peimine being significantly higher than peiminine, and the combination group was significantly increased compared with the single group. This trend was the same for anti-inflammatory effects: 100.0 *μ*g/mL combination >100.0 *μ*g/mL peimine >100.0 *μ*g/mL peiminine. Compared to the normal combination group, *I*_max_ of peimine and peiminine increased in the model combination group. This might be due to the significant inhibitory effects of peimine and peiminine on cytokine secretion, thus increasing the uptake of cellular drugs in the model group.

## 5. Conclusions

The UPLC-MS/MS method established in this study was found to be selective and stable for the simultaneous determination of peimine and peiminine in A549 cells. The model group could uptake more than the normal group. Compared with the single group, the combination group showed a higher untake. The combination group's anti-inflammatory effects were improved effectively. In this paper, the uptake of peimine and peiminine in cellular was systematically investigated, and the scientific date can provide a basis for the research of anti-inflammatory mechanisms and may be useful for the further clinical practice of of peimine and peiminine.

## Figures and Tables

**Table 1:** Pharmacodynamic study: effects of main components of Thunberg fritillary bulb in different concentrations on inflammatory cell model.

Group					
	IL-8	MMP-9		TIMP-1	TIMP-1/MMP-9
Blank group	137.51 ± 10.40^*∗∗*^	23.18 ± 2.87^*∗∗*^		6118.79 ± 195.13^*∗∗*^	262.05 ± 34.64^*∗∗*^
Model group	2360.38 ± 116.63	1336.91 ± 62.88		18125.41 ± 1938.48	13.32 ± 1.66
12.5 *μ*g/mL peimine	2733.26 ± 495.81^*∗*^	914.58 ± 25.88^*∗∗*^		17902.34 ± 2687.66	19.19 ± 2.81
25.0 *μ*g/mL peimine	2266.70 ± 180.83	623.25 ± 20.38^*∗∗*^		11763.68 ± 1872.61^*∗*^	18.56 ± 3.33
50.0 *μ*g/mL peimine	1873.92 ± 269.34^*∗∗*^	349.04 ± 15.31^*∗∗*^		14262.72 ± 8942.73	39.90 ± 24.53^*∗*^
100.0 *μ*g/mL peimine	1461.52 ± 111.69^*∗∗*^	116.02 ± 10.22^*∗∗*^		5857.64 ± 202.47^*∗∗*^	49.84 ± 5.31^*∗*^
12.5 *μ*g/mL peiminine	2918.69 ± 513.74^*∗*^	774.87 ± 21.99^*∗∗*^		8998.30 ± 443.72^*∗∗*^	11.40 ± 0.85
25.0 *μ*g/mL peiminine	3272.47 ± 436.04^*∗∗*^	735.03 ± 51.64^*∗∗*^		10039.44 ± 1358.27^*∗∗*^	13.39 ± 1.64
50.0 *μ*g/mL peiminine	2750.58 ± 404.06	377.34 ± 12.80^*∗∗*^		9334.98 ± 3243.91^*∗∗*^	24.25 ± 8.42
100.0 *μ*g/mL peiminine	2315.88 ± 397.02	213.47 ± 10.54^*∗∗*^		8333.69 ± 1425.33^*∗∗*^	38.47 ± 6.22^*∗*^
25.0 *μ*g/mL combination	2668.04 ± 303.96^*∗∗*^	458.17 ± 28.94^*∗∗*^		13314.78 ± 1411.56	28.48 ± 2.82
50.0 *μ*g/mL combination	2143.58 ± 275.69	249.15 ± 18.28^*∗∗*^		9199.63 ± 506.44^*∗∗*^	36.18 ± 4.48^*∗*^
100.0 *μ*g/mL combination	1870.99 ± 251.36^*∗∗*^	171.68 ± 9.84^*∗∗*^		7222.64 ± 353.93^*∗∗*^	41.23 ± 2.78^*∗*^
200.0 *μ*g/mL combination	1268.63 ± 119.03^*∗∗*^	101.58 ± 13.08^*∗∗*^		5316.92 ± 208.86^*∗∗*^	51.29 ± 4.92^*∗*^

**Table 2:** Regression equation, linearity, and LOQ.

Component	Regression equation	Linear range (ng/mL)	LOQ (ng/mL)	LOD (ng/mL)	Correlation coefficient (*r*)
Peimine	*y* = 0.0073*X* + 0.017	0.4008∼2004	0.4008	0.1982	0.9979
Peiminine	*y* = 0.0082*X* + 0.007	0.2840∼1420	0.2840	0.0951	0.9999

**Table 3:** Extraction recovery and matrix effect at low, medium, and high concentration levels (mean ± SD, *n* = 6).

Component	Spiked (ng/mL)	Recovery		Matrix effect	
		Mean (%)	RSD (%)	Mean (%)	RSD (%)
Peimine	2.004	89.81 ± 0.80	0.89	106.55 ± 0.26	0.24
	20.04	102.04 ± 3.07	3.01	95.05 ± 2.20	2.31
	200.4	83.85 ± 2.86	3.41	109.25 ± 1.76	1.61

Peiminine	1.42	113.67 ± 2.32	2.04	111.29 ± 1.80	1.62
	14.2	84.89 ± 3.54	4.17	96.31 ± 4.96	5.15
	142	92.04 ± 4.49	4.88	95.44 ± 4.79	5.02

**Table 4:** Intraday and interday accuracy and precision.

Component	Spiked (ng/mL)	Intraday			Interday		
		Measured (ng/mL)	Accuracy (RE%)	Precision (RSD%)	Measured (ng/mL)	Accuracy (RE%)	Precision (RSD%)
Peimine	2.004	2.02 ± 0.13	0.55	6.24	2.01 ± 0.08	0.38	4.33
	20.04	20.02 ± 0.81	−0.08	4.05	20.28 ± 1.25	1.19	6.19
	200.4	199.87 ± 4.28	−0.26	2.14	199.96 ± 9.87	−0.22	4.94

Peiminine	1.42	1.45 ± 0.06	1.76	3.91	1.46 ± 0.11	3.05	7.73
	14.2	14.39 ± 0.96	1.34	6.73	14.58 ± 0.53	2.69	3.63
	142	141.28 ± 5.01	−0.51	3.55	144.61 ± 7.76	1.84	5.36

**Table 5:** Stability investigated for peimine and peiminine under different conditions.

Component	Spiked (ng/mL)	Short-time stability		Long-time stability		Freeze-thaw (three cycles)	
		Measured (ng/mL)	Accuracy (%)	Measured (ng/mL)	Accuracy (%)	Measured (ng/mL)	Accuracy (%)
Peimine	2.004	2.06 ± 0.13	2.96	2.07 ± 0.09	3.46	1.99 ± 0.17	−0.69
	20.04	20.55 ± 1.17	2.52	20.39 ± 0.92	1.74	20.62 ± 1.37	2.89
	200.4	200.81 ± 1.31	0.21	200.69 ± 2.84	0.15	200.84 ± 3.22	0.22

Peiminine	1.42	1.46 ± 0.07	2.46	1.44 ± 0.07	1.29	1.43 ± 0.06	0.94
	14.2	14.52 ± 0.57	2.27	14.42 ± 0.79	1.57	14.53 ± 0.49	2.35
	142	144.12 ± 5.57	1.49	143.02 ± 3.70	0.71	141.19 ± 2.97	−0.57

**Table 6:** Incubation time and intracellular uptake of peimine and peiminine ($\bar{x}\pm \text{SD},n=3$) (ng/*μ*g).

Time (min)	Peimine				Peiminine			
	Normal (single)	Model (single)	Normal (combination)	Model (combination)	Normal (single)	Model (single)	Normal (combination)	Model (combination)
10	374.18 ± 55.05	265.35 ± 39.31	452.03 ± 40.11	324.10 ± 45.60	265.97 ± 40.08	322.31 ± 31.42	277.12 ± 41.25	322.69 ± 60.59
30	487.84 ± 42.89	499.99 ± 28.16	537.76 ± 45.79	378.60 ± 20.04	321.18 ± 46.23	340.59 ± 19.31	379.74 ± 30.28	353.65 ± 45.16
45	598.40 ± 32.29	722.54 ± 61.64	614.98 ± 75.52	556.84 ± 44.22	363.03 ± 34.37	460.30 ± 38.29	544.43 ± 93.35	495.07 ± 72.30
60	677.41 ± 89.83	929.41 ± 77.31	731.64 ± 74.53	731.46 ± 56.16	449.07 ± 39.65	622.72 ± 72.70	584.93 ± 96.59	502.99 ± 55.31
90	719.23 ± 66.10	1123.33 ± 118.71	805.51 ± 90.48	1178.28 ± 95.34	437.35 ± 51.59	576.36 ± 48.81	612.09 ± 34.75	650.82 ± 51.18
120	1667.58 ± 109.83	1823.28 ± 97.18	1064.93 ± 92.15	1224.03 ± 130.88	429.70 ± 27.15	542.43 ± 27.15	660.64 ± 135.82	567.29 ± 47.15
180	1544.43 ± 112.84	1796.39 ± 97.58	2534.09 ± 209.61	1710.40 ± 125.68	386.52 ± 25.80	509.05 ± 39.61	705.36 ± 63.06	745.71 ± 90.01
240	1489.08 ± 212.90	1682.04 ± 178.46	2933.14 ± 126.76	3669.97 ± 109.91	328.83 ± 32.15	473.62 ± 29.41	765.37 ± 99.30	1101.01 ± 106.62
360	1451.28 ± 59.83	1576.08 ± 44.72	2893.19 ± 195.27	3596.86 ± 203.03	315.79 ± 38.62	420.19 ± 34.23	702.91 ± 53.12	1041.95 ± 115.87
480	1405.24 ± 55.29	1550.79 ± 139.51	2627.98 ± 127.57	3550.15 ± 160.75	306.45 ± 15.12	406.67 ± 20.38	675.99 ± 102.03	974.98 ± 56.96

**Table 7:** Release time and intracellular uptake of peimine and peiminine ($\bar{x}\pm \text{SD},n=3$) (ng/*μ*g).

Time (min)	Peimine				Peiminine			
	Normal (single)	Model (single)	Normal (combination)	Model (combination)	Normal (single)	Model (single)	Normal (combination)	Model (combination)
0	1660.54 ± 121.83	1907.38 ± 100.05	3060.96 ± 209.19	3547.61 ± 208.20	474.85 ± 42.71	608.14 ± 51.74	778.36 ± 68.96	1009.38 ± 103.26
10	259.63 ± 30.98	161.63 ± 19.81	575.85 ± 71.54	529.40 ± 58.09	80.37 ± 10.87	77.89 ± 9.60	69.51 ± 8.13	86.17 ± 13.45
30	46.33 ± 5.95	45.07 ± 7.01	75.26 ± 13.68	137.79 ± 12.15	35.61 ± 3.58	49.76 ± 4.08	36.42 ± 4.57	48.86 ± 7.62
45	28.32 ± 3.84	31.11 ± 3.29	34.04 ± 5.08	35.41 ± 4.56	24.58 ± 2.05	30.70 ± 1.79	7.30 ± 1.20	9.43 ± 1.42
60	14.11 ± 1.42	22.81 ± 3.00	24.27 ± 2.50	29.08 ± 1.56	6.83 ± 0.56	17.06 ± 1.44	6.50 ± 0.66	4.84 ± 0.76
90	8.81 ± 1.31	16.44 ± 2.56	25.84 ± 2.93	18.65 ± 4.55	4.81 ± 1.40	6.21 ± 0.65	4.55 ± 0.47	4.66 ± 0.49
120	6.63 ± 0.80	11.47 ± 1.51	9.71 ± 1.08	16.67 ± 1.94	4.32 ± 1.83	3.61 ± 0.29	3.51 ± 0.74	3.37 ± 0.19
180	5.06 ± 0.77	8.90 ± 0.97	7.67 ± 1.82	13.94 ± 1.62	2.95 ± 0.38	3.57 ± 0.39	2.93 ± 0.18	3.31 ± 0.25
240	4.72 ± 0.44	4.53 ± 0.73	6.63 ± 0.99	8.15 ± 1.00	2.64 ± 0.63	3.32 ± 0.22	2.74 ± 0.45	3.29 ± 0.30
360	2.17 ± 0.25	3.81 ± 0.69	6.06 ± 1.21	6.80 ± 0.87	2.58 ± 0.29	2.47 ± 0.30	2.16 ± 0.20	3.17 ± 0.19
480	2.08 ± 0.26	3.65 ± 0.44	5.27 ± 0.92	6.54 ± 0.55	2.51 ± 0.36	2.40 ± 0.36	1.86 ± 0.41	2.74 ± 0.46
720	1.10 ± 0.11	2.13 ± 0.22	2.25 ± 0.43	3.95 ± 0.45	1.99 ± 0.33	2.04 ± 0.18	0.97 ± 0.20	2.43 ± 0.37
1440	1.14 ± 0.07	0.85 ± 0.12	1.98 ± 0.27	2.77 ± 0.77	1.18 ± 0.16	1.22 ± 0.19	0.81 ± 0.24	1.11 ± 0.16

## Data Availability

The data used to support the findings of this study are included within the article.
